# A comprehensive overview of recent developments on the mechanisms and pathways of ferroptosis in cancer: the potential implications for therapeutic strategies in ovarian cancer

**DOI:** 10.20517/cdr.2023.49

**Published:** 2023-08-11

**Authors:** Hiroshi Kobayashi, Chiharu Yoshimoto, Sho Matsubara, Hiroshi Shigetomi, Shogo Imanaka

**Affiliations:** ^1^Department of Gynecology and Reproductive Medicine, Ms.Clinic MayOne, Kashihara 634-0813, Japan.; ^2^Department of Obstetrics and Gynecology, Nara Medical University, Kashihara 634-8522, Japan.; ^3^Department of Obstetrics and Gynecology, Nara Prefecture General Medical Center, Nara 630-8581, Japan.; ^4^Department of Medicine, Kei Oushin Clinic, Nishinomiya 663-8184, Japan.; ^5^Department of Gynecology and Reproductive Medicine, Aska Ladies Clinic, Nara 634-0001, Japan.

**Keywords:** Ferroptosis, glutaminolysis, glycolysis, metabolic vulnerability, ovarian cancer, pentose phosphate pathway

## Abstract

Cancer cells adapt to environmental changes and alter their metabolic pathways to promote survival and proliferation. Metabolic reprogramming not only allows tumor cells to maintain a reduction-oxidation balance by rewiring resources for survival, but also causes nutrient addiction or metabolic vulnerability. Ferroptosis is a form of regulated cell death characterized by the iron-dependent accumulation of lipid peroxides. Excess iron in ovarian cancer amplifies free oxidative radicals and drives the Fenton reaction, thereby inducing ferroptosis. However, ovarian cancer is characterized by ferroptosis resistance. Therefore, the induction of ferroptosis is an exciting new targeted therapy for ovarian cancer. In this review, potential metabolic pathways targeting ferroptosis were summarized to promote anticancer effects, and current knowledge and future perspectives on ferroptosis for ovarian cancer therapy were discussed. Two therapeutic strategies were highlighted in this review: directly inducing the ferroptosis pathway and targeting metabolic vulnerabilities that affect ferroptosis. The overexpression of SLC7A11, a cystine/glutamate antiporter SLC7A11 (also known as xCT), is involved in the suppression of ferroptosis. xCT inhibition by ferroptosis inducers (e.g., erastin) can promote cell death when carbon as an energy source of glucose, glutamine, or fatty acids is abundant. On the contrary, xCT regulation has been reported to be highly dependent on the metabolic vulnerability. Drugs that target intrinsic metabolic vulnerabilities (e.g., GLUT1 inhibitors, PDK4 inhibitors, or glutaminase inhibitors) predispose cancer cells to death, which is triggered by decreased nicotinamide adenine dinucleotide phosphate generation or increased reactive oxygen species accumulation. Therefore, therapeutic approaches that either directly inhibit the xCT pathway or target metabolic vulnerabilities may be effective in overcoming ferroptosis resistance. Real-time monitoring of changes in metabolic pathways may aid in selecting personalized treatment modalities. Despite the rapid development of ferroptosis-inducing agents, therapeutic strategies targeting metabolic vulnerability remain in their infancy. Thus, further studies must be conducted to comprehensively understand the precise mechanism linking metabolic rewiring with ferroptosis.

## INTRODUCTION

Epithelial ovarian cancer has a poor prognosis because of limited early screening methods and high recurrence rates^[1]^. Modern approaches include debulking surgery, platinum/taxane chemotherapy, angiogenesis inhibitors (e.g., bevacizumab), drugs targeting poly(ADP-ribose) polymerase (i.e., PARP inhibitors), and drugs targeting the dysfunctional immune system (i.e., immune checkpoint inhibitors) depending on the histological type and tumor stage^[2]^. Thus, establishing more effective treatment options is necessary as ovarian cancer often recurs and becomes resistant to chemotherapy.

High-grade serous ovarian cancer (HGSC) is a common and aggressive subtype of epithelial ovarian cancer^[3]^. Clear cell carcinoma (CCC) of the ovary, the second most common histological type in Japan, has distinctive clinical behavior, biological function, and molecular characteristics^[3]^. HGSC arises from the implantation of fallopian tube epithelial cells, and it is characterized by TP53 (p53) mutations associated with enhanced genomic instability^[3]^. p53 mutation may promote hypoxia-induced genomic instability, leading to the activation of pro-oncogenic signaling such as hypoxia-inducible factor (HIF) 1^[4]^. Furthermore, fallopian tube epithelial cells are constantly exposed to potentially toxic constituents [e.g., labile iron and reactive oxygen species (ROS)] derived from retrograde menstrual reflux even before the accumulation of somatic or oncogenic mutations^[5]^. The iron content determined in HGSC was almost five times higher than that for normal ovarian cells^[6]^. HGSC exhibits an increased expression level of the iron import transferrin receptor 1 and a decreased expression level of the cellular iron exporter ferroportin, thereby enhancing the intracellular iron pool^[7]^. In addition, based on previous reports, CCC derived from endometriosis could increase CCC cell proliferation via iron supplied by the surrounding endometriosis^[8]^. Rapidly proliferating cancer cells have a unique phenotype of iron metabolism, which increases iron supply and decreases iron loss^[9]^. Apart from iron, such cancer cells also require a continuous supply of adequate nutrients and oxygen available within the tumor microenvironment to generate their own bioenergetics (e.g., glucose, glutamine, cysteine, ATP, and fatty acid) and macromolecules (e.g., proteins, lipids, and nucleic acids)^[10]^. Glycolysis, mitochondrial oxidative phosphorylation (OXPHOS), glutaminolysis, and fatty acid synthesis are the major pathways of energy metabolism. Ovarian cancer cells switch from OXPHOS to aerobic glycolysis to adapt to environmental changes^[11]^ through HIF-mediated metabolic reprogramming^[12]^.

Cancer cells acquire a diverse range of metabolic flexibility and plasticity as an adaptation to ever-changing nutritional microenvironments during tumor evolution^[13]^. However, accelerated energy metabolism that supports the enhanced proliferation rate further increases iron demand, oxidative stress, and susceptibility to tumor cell death^[14]^. Ferroptosis is a recognized form of regulated cell death associated with iron and ROS accumulation, which serves as a vital component of various processes in ovarian cancer^[7,15]^. Under nutrient-deprived conditions, cancer cells depend on optimal metabolic pathways for survival. This metabolic reprogramming creates an addiction to intracellular and extracellular nutrients (i.e., strong dependencies on nutrients), which may result in acquired resistance to cell death^[13]^. Metabolic flexibility and nutrient addiction are critical for cell fate determination. Therefore, forced alterations in metabolic pathways may have great application potential in tumor-targeted therapy. Thus, this review aims to summarize the regulation of ferroptosis evasion and its crosstalk with multiple cellular metabolic pathways in a variety of cancers and to discuss research perspectives, particularly therapeutic strategies targeting ovarian cancer.

## METABOLIC FLEXIBILITY, PLASTICITY, AND VULNERABILITY IN CANCER CELLS

Cells have evolved elaborate metabolic flexibility to control cell survival, growth, and defense against oxidative stress for adaptation to ever-changing aerobic or anaerobic environments^[16]^. Glucose is the major energy source for eukaryotic cells, and it plays a critical role in redox (reduction/oxidation) homeostasis^[10]^. Glucose is transported into cells via transmembrane proteins [e.g., glucose transporter (GLUT)] and metabolized through glycolysis and the pentose phosphate pathway (PPP), an early diverging branch of glycolysis^[17]^. The internalized glucose molecule is initially converted to glucose 6-phosphate (G6P) by hexokinase, and then it produces pyruvate through glycolysis as well as abundant reduced nicotinamide adenine dinucleotide phosphate (NADPH) and ribose 5-phosphate through the PPP^[17]^. NADPH serves as a cofactor for glutathione reductase and maintains the active state of antioxidants by converting oxidized glutathione [i.e., glutathione disulfide (GSSG)] to glutathione (GSH)^[14]^. Ribose-5-phosphate is necessary for nucleic acid synthesis^[14]^. Pyruvate is metabolized to lactate in the cytosol or converted to acetyl coenzyme A (acetyl-CoA) in the mitochondria to fuel the tricarboxylic acid (TCA) cycle, enhance OXPHOS, and generate ATP^[18,19]^. Under hypoxic conditions, pyruvate is converted into lactate during glycolysis^[20]^.

Cancer cells adapt to the tumor environment by reprogramming various metabolic pathways to meet their high-energy demands. The upregulation of specific nutrient transporters increases cellular entry of glucose and amino acids^[21]^. However, the generation of ATP as an energy source in the mitochondria induces oxidative stress caused by increased ROS production, resulting in cell death^[11,22,23]^. In mitigating oxidative stress, glucose metabolism is rewired to the PPP that operates parallel to glycolysis to maintain redox homeostasis by generating ribose-5-phosphate and NADPH^[11]^. Cancer cells prefer aerobic glycolysis over OXPHOS to support their proliferation, which is known as the Warburg effect^[22,23]^. Pyruvate is irreversibly converted to acetyl-CoA by pyruvate dehydrogenase (PDH) in the mitochondria. Pyruvate dehydrogenase kinase (PDK) inhibits PDH activity^[24]^. Thus, PDK suppresses the metabolic shift from glycolysis to the TCA cycle by downregulating PDH, thereby reducing mitochondrial ROS production and suppressing cell death^[24,25]^. Furthermore, cancer cells upregulate the PPP, an early diverging branch of glycolysis, thereby promoting NADPH production and exerting antioxidant defenses^[26]^. Cancer cells not only switch from OXPHOS to lactate-dependent energy-generating pathways, but also upregulate antioxidant-related genes [e.g., NF-E2-related factor 2 (Nrf2)] to overcome a wide range of oxidative stress^[27]^. Furthermore, NRF2 accelerates cellular redox homeostasis by upregulating the transcriptional regulation of multiple NADPH-generating enzyme genes (e.g., G6PD)^[26]^. Cancer cells rely not only on glucose, but also on glutamine for their energy demands^[25]^. Glutaminase is a critical enzyme that converts glutamine to glutamate in the mitochondria^[28]^. Glutamate contributes to GSH synthesis and maintains redox homeostasis through reduced ROS generation^[29]^. Apart from glycolysis in cancer cells, glutaminolysis (i.e., a series of biochemical reactions by which glutamine is lysed to glutamate) is another main pillar for energy production, including the conversion of glutamine to α-ketoglutarate (αKG), reaction steps of the citric acid cycle and malate aspartate shuttle, and the conversion of malate to pyruvate and lactate. Glutaminolysis increases OXPHOS and compensates for the energy deficiency in glycolytic cancer cells^[25]^.

In addition, altered metabolism or metabolic rewiring facilitates the adaptation of cancer cells to changing external environments, and this adaptation may render certain nutrients indispensable, a process known as nutrient addiction^[15]^. Glutamine^[30]^ and cystine addiction have been found in renal cell carcinoma^[31]^, breast cancer^[32]^, and non-small cell lung cancer^[15,33]^. HGSC and CCC are highly sensitive to cystine-deprived death, exhibiting a cystine addiction phenotype^[15,34,35]^. In addition, heterogenous tumors are composed of multiple subpopulations associated with different proliferative and malignant potentials^[36]^. For example, specific tumor types have different metabolic features; primary tumors build biomass such as glucose to sustain their high proliferative demands; metastatic tumors rely on pyruvate, glutamine, and lipid metabolism, and cancer stem cells depend on mitochondrial metabolisms such as OXPHOS and aerobic glycolysis^[12,37]^. Such a nutrient addiction can be targeted for therapy because many cancer cells have limited energy and nutrient flexibility^[25,26,38]^.

## THE ROLE OF FERROPTOSIS IN CANCER

Eukaryotic cells produce energy in the form of ATP and generate ROS as a byproduct. Such cells have evolved an array of antioxidant mechanisms, such as the thiol system, to combat oxidative stress, including nitric oxide, carbon monoxide, and hydrogen (per)sulfide^[27]^. Cancer cells have also evolved the antioxidant defense system to protect themselves from excess ROS, but oxidative stress exceeding the antioxidant defense mechanism leads to cell death. Cell death is divided into two forms, namely, accidental cell death and regulated cell death^[39]^. The latter is subdivided into apoptotic and non-apoptotic cell death. The non-apoptotic cell death includes autophagy, ferroptosis, pyroptosis, and necroptosis^[40]^. Ferroptosis is characterized by the iron-dependent accumulation of excessive ROS and lipid peroxides, leading to cell death^[15,35,41-44]^. Excess iron in ovarian cancer amplifies free oxidative radicals and drives the Fenton reaction, thereby inducing ferroptosis^[7,15]^. However, HGSC is characterized by ferroptosis resistance because it can acquire sufficient antioxidant capacity. For example, Nrf2, a representative antioxidant gene, activates the transcription of ferritin heavy chain 1 (a protein involved in iron storage) and heme oxygenase-1 (a protein involved in heme breakdown) to reduce labile iron and regulate iron metabolism and oxidative stress^[15,45]^. These antioxidants block ferroptosis by limiting cellular oxidative stress and lipid peroxidation.

In general, ferroptosis is regulated by membrane transporter expression, metabolic flexibility, and nutrient dependency. First, solute carrier family 7 member 11 (SLC7A11), commonly known as xCT, is a cystine/glutamate antiporter^[19]^. The xCT pathway plays an important role in antioxidant defense by transporting extracellular cystine into cells and converting cystine to cysteine for GSH biosynthesis and ROS detoxification^[16,46-48]^. GSH is synthesized from three constituent amino acids, namely, cysteine, glycine, and glutamic acid, with cysteine being a rate-limiting precursor. Glutathione peroxidase 4 (GPX4) utilizes GSH to convert lipid hydroperoxides into nontoxic lipid alcohols, thereby preventing ferroptosis^[19]^. HGSCs are characterized by xCT overexpression along with the activation of GSH biosynthesis^[48]^. Such a metabolic landscape suggests that HGSC depends on cystine uptake to counteract high levels of iron-dependent oxidative stress and maintain redox homeostasis, thereby preventing ferroptosis-induced cell death^[16]^. Therefore, the inhibition of xCT-mediated cystine transport, cystine depletion, limited cysteine biosynthesis, impaired GSH synthesis, or inactivation of GPX4 can induce ferroptosis^[49]^. During ferroptosis, the following breakdown products of lipid peroxides are formed, for example, malondialdehyde (MDA), 4-hydroxynonenal, 4-hydroxyhexenal, and 4-oxo-nonenal, and oxidized and modified proteins^[50]^. High serum levels of MDA have been reported in patients with ovarian cancer compared with healthy women^[51]^, indicating the occurrence of ferroptosis. Such breakdown products may be evaluated as potential surrogate biomarkers for ferroptosis.

Second, ferroptosis is significantly influenced by metabolic flexibility and nutrient dependency. A large amount of NADPH generated via the PPP is consumed to synthesize cysteine and GSH^[26]^. Thus, cancer cells must evolve mechanisms to cope with NADPH depletion. For example, cancer cells upregulate the expression level of the PPP^[26]^, NRF2^[52]^, and isocitrate dehydrogenase 1 (IDH1)^[48]^ as backup mechanisms for NADPH production. Nrf2 enhances the cellular antioxidant defense through NADPH regeneration^[53]^. NADPH is also produced during the NADP^+^-dependent conversion of isocitrate to alpha-ketoglutarate (αKG) by IDH1^[48]^. These backup mechanisms facilitate the survival and proliferation of cancer cells^[26,48,52]^. Therefore, drugs that block the uptake of specific nutrients may provide potential therapeutic opportunities to kill cancer cells that rely on the same metabolic pathway^[54]^. Therapeutic strategies targeting nutrient addiction or metabolic vulnerabilities may induce ferroptosis and inhibit tumor growth. Strategies targeting metabolic vulnerabilities will not only eliminate backup systems to prevent ferroptosis^[49]^, but also receive considerable attention in cancer therapeutics^[54]^.

## CURRENT UNDERSTANDING OF METABOLIC PATHWAYS INVOLVED IN FERROPTOSIS

**Figure 1:**
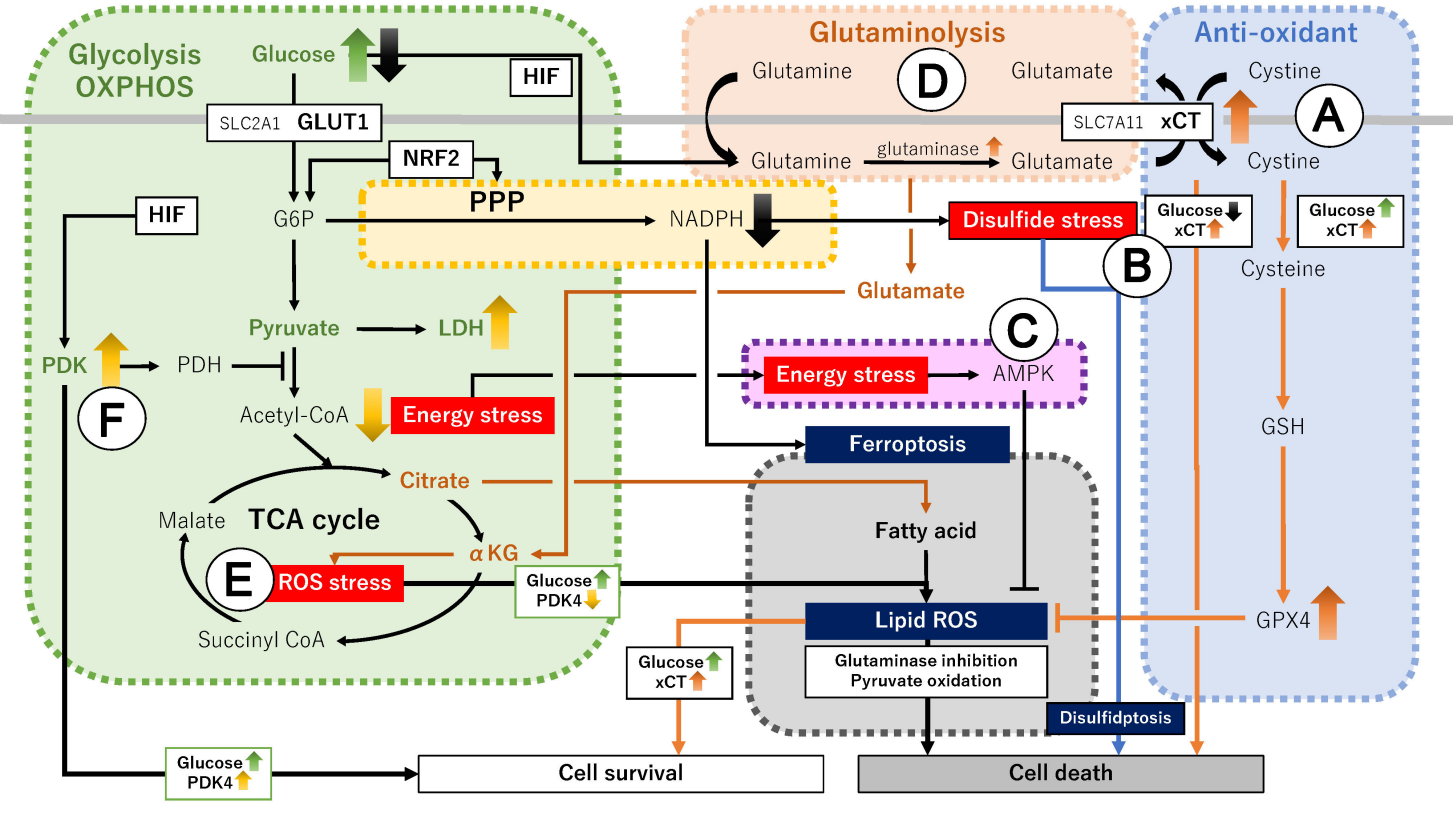
Metabolic pathways involved in ferroptotic cell death. Green, yellow, brown, purple, blue and gray boxes indicate glycolysis and OXPHOS, PPP, glutaminolysis, energy stress, antioxidant, and ferroptosis pathways, respectively. αKG: α-ketoglutarate; AMPK: AMP-activated protein kinase; G6P: glucose 6-phosphate; GLUT1/SLC2A1: glucose transporter 1; GPX4: glutathione peroxidase 4; GSH: glutathione; HIF: hypoxia-inducible factor; LDH: lactate dehydrogenase; NADPH: nicotinamide adenine dinucleotide phosphate; NRF2: NF-E2-related factor 2; OXPHOS: oxidative phosphorylation; PDH: pyruvate dehydrogenase; PDK: pyruvate dehydrogenase kinase; PPP: pentose phosphate pathway; ROS: reactive oxygen species; SLC7A11: solute carrier family 7 member 11; TCA: tricarboxylic acid; xCT: cystine/glutamate antiporter SLC7A11.

Glucose, glutamine, and fatty acids are major metabolic fuels to meet nutritional demands^[16]^. Tumor cells receive energy supplies from unique metabolic pathways such as glycolysis, PPP, glutaminolysis, and OXPHOS; upregulate NADPH and GSH production; and downregulate ROS production to control ferroptosis^[14]^. First, we summarized the mechanism by which glucose regulates ferroptosis in the context of energy stress^[18]^. Under intact PPP and glycolysis with ample glucose and amino acid supply, NADPH can support the xCT‐mediated cystine uptake^[14,48]^. Free cystine accumulated in cancer cells forms a water-insoluble toxic crystal, often leading to cell damage (i.e., disulfide stress)^[48]^. Cystine crystals induce ROS production through increased disulfide stress and promote oxidative stress reactions within cancer cells^[48]^. NADPH can suppress intracellular cystine-dependent disulfide stress by converting cystine into cysteine for GSH synthesis^[14,48]^. Thus, the PPP‐generated NADPH rescues xCT-overexpressing cancer cells (xCT^high^ cancer cells) from ferroptosis, demonstrating that xCT^high^ cancer cells become highly dependent on the glucose-PPP pathway (i.e., NADPH addiction) to inhibit ferroptosis [Figure 1A].

On the contrary, peritumoral angiogenesis cannot keep up with rapidly proliferating lesions, resulting in regions of low blood supply. Such tumor cells must survive under glucose deprivation-induced metabolic stress conditions^[14,55]^. Glucose starvation decreases carbon flux, which leads to the depletion of PPP and NADPH, inhibition of the conversion of cystine to cysteine, and marked accumulation of cystine, a disulfide molecule^[46,56]^. xCT-mediated cystine uptake suppresses ferroptosis under conditions with sufficient glucose supply; however, its effect might be limited under glucose starvation, suggesting that glucose starvation may be associated with increased disulfide stress and ferroptosis^[48,56]^ [Figure 1B]. Sufficient glucose supply prevents ferroptosis in cancer cells, whereas glucose deprivation promotes cell death, indicating that cystine addiction determines the survival or death of cancer cells^[48]^. In addition, limited glucose supply results in cell death in xCT^high^ cancer cell lines, including breast, cervical, kidney, and glioblastoma^[48,57]^. Further experiments validated that the downregulation of xCT expression suppresses the promoting effect of cancer cell death under nutritional deficiency^[58]^. However, persistent chronic glucose starvation in surviving ovarian cancer cells results in phenotypic changes through metabolic plasticity, leading to the acquisition of drug resistance and cancer relapse^[59]^.

Second, we summarized the mechanism by which energy stress positively and negatively regulates ferroptosis. Recent research has focused on a potential link between AMP-activated protein kinase (AMPK), a critical sensor of cellular energy status, and ferroptosis^[55]^. Energy stress caused by glucose starvation activates AMPK^[55]^, and the activated AMPK circumvents metabolic stress by restoring energy balance^[60]^. AMPK inactivates lipogenic genes, such as acetyl-CoA carboxylase (ACC), and it could inhibit ferroptosis^[55]^ [Figure 1C]. However, conflicting data have also been reported, that is, AMPK promotes ferroptosis^[61]^. AMPK triggers the formation of a beclin 1 (BECN1)-SLC7A11 complex via BECN1 phosphorylation^[61]^. The binding of BECN1 to SLC7A11, a core component of xCT, inhibits the xCT function and promotes ferroptosis^[62]^, indicating that BECN1 is a key ferroptosis inducer^[63]^. In xCT- and BECN1-overexpressing cancer cells, AMPK phosphorylates BECN1, induces binding to SLC7A11, and promotes ferroptosis by directly blocking xCT activity^[64]^. Therefore, AMPK either inhibits ferroptosis in response to glucose starvation or promotes ferroptosis by blocking xCT activity. The expression level of ACC or BECN1 in AMPK-activated cancer cells may be critical in determining cell fate. AMPK has also been reported to promote tumor growth by alleviating energy stress or to suppress tumor growth by inhibiting key metabolic pathways such as glucose, glutamine, and fatty acid biosynthesis^[65]^. These findings suggest that energy stress exerts inhibitory and promoting effects during ferroptosis, but the detailed mechanism remains unclear. Recent studies have reported that AMPK-activating drugs may affect metabolic plasticity^[66]^.

Third, we summarized the mechanism by which glutamine metabolism is involved in ferroptosis, particularly on xCT. Under glucose deprivation, tumor cells rely on an alternative energy-generating pathway such as glutaminolysis^[25,67]^ [Figure 1D]. In meeting the high-energy demand, tumor cells express high levels of glutamine transporters and glutamine synthase, often resulting in glutamine addiction^[13,25,29]^. Glutamate is converted to αKG, an intermediate in the TCA cycle in glutaminolysis, which activates the TCA cycle, although much remains exported by xCT in exchange for extracellular cystine^[16]^. Therefore, the loss of cellular glutamate could reduce αKG production and suppress TCA cycle activities, thereby leading to reduced lipid ROS levels and decreased ferroptosis sensitivity^[16]^. This process may be a characteristic of xCT^high^ cancer cells. Over time, xCT imports extracellular cystine in exchange for intracellular glutamate release. Glutaminolysis supplies cancer cells with adequate glutamate to maintain extracellular cystine uptake^[16,26,46]^; however, the accumulated excess intracellular cystine induces disulfide stress, leading to the overproduction of ROS and gradually promoting ferroptosis^[16]^. Furthermore, sustained cystine uptake leads to extracellular cystine depletion, suppressing glutamate export and promoting intracellular glutamate accumulation. Glutamate may be converted to αKG to maintain TCA cycle function and subsequently enhance the production of lipid ROS to induce ferroptosis^[16,68]^. In particular, high-OXPHOS ovarian cancer cells were demonstrated to utilize glutamine, reconfirming the importance of glutamine^[69]^. Therefore, ferroptosis sensitivity may depend on the glucose starvation status, the level of intracellular NADPH, αKG, or glutamine and extracellular cystine, and the expression level of xCT in each cancer cell^[16,26,46,58]^.

In addition, energy stress such as the depletion and overproduction of ATP in cancer cells determines cell fate. The mitochondria are the main sites for ATP generation through OXPHOS, producing ROS as a byproduct of cellular metabolism^[13,70]^. In cysteine-depleted cancer cells, the TCA cycle activated by αKG induces cellular lipid ROS levels, thereby promoting cell death^[71]^ [Figure 1E]. The moderate suppression of the TCA cycle and OXPHOS reduces mitochondrial ROS production and inhibits cell death^[71]^. However, extreme ATP depletion (i.e., energy deprivation) induces cell death. That is, energy stress can induce cell death by ATP depletion (i.e., energy deprivation) and ATP overproduction (e.g., excess ROS generation caused by increased αKG). In addition, homologous recombination-deficient cancer cells require ATP for poly(ADP-ribose) polymerase (PARP)-dependent DNA repair mechanisms used in ovarian cancer treatment, demonstrating that ATP causes sensitivity to PARP inhibitors^[72]^.

Fourth, we summarized the mechanism by which metabolic reprogramming of glycolysis and OXPHOS regulates ferroptosis. Hypoxia is the major cause of the rapid growth of cancer cells. Metabolic reprogramming is essential for the adaptation of cancer cells to a hypoxic microenvironment^[73]^. HIF-1α overexpressed under hypoxic conditions contributes to aggressive phenotypes in tumor cells^[13]^. There are two types of energy production from glucose: glycolysis and mitochondrial OXPHOS^[74]^. HIF-1 activates glycolysis-related genes, including pyruvate dehydrogenase kinase 1 (PDK1), increasing the conversion of glucose to pyruvate and subsequently to lactate by inactivating PDH, which is considered critical for metabolic adaptation to hypoxia^[74]^ [Figure 1F]. PDK4 has been reported to block a metabolic switch from glycolysis to predominantly fatty acid synthesis and contribute to ferroptosis resistance in certain cancer cells, such as pancreatic ductal adenocarcinoma (PDAC) cells^[18,19]^. PDK1 can also mediate a metabolic shift from mitochondrial OXPHOS to glycolysis and increase the proliferation and angiogenesis in ovarian cancer xenografts^[75]^. Therefore, the Warburg effect has a profound impact on ferroptosis pathways through PDK-dependent metabolic switches or metabolic adaptations in cancer.

Fifth, we focused on the role of the tumor suppressor p53 in the pathophysiological process of ferroptosis in ovarian cancer. p53 induces essential biological processes such as cell cycle arrest, senescence, DNA repair, apoptosis, autophagy, and the reprogramming of cellular metabolism^[38]^. p53 has been reported to regulate various metabolic pathways and promote metabolic reprogramming to induce drug resistance and metastasis^[38]^. In particular, p53 inhibits SLC7A11 expression and reduces glutathione synthesis, making cancer cells susceptible to oxidative damage and sensitive to iron by increasing lipid peroxide levels^[32,76,77]^. In a previous study, Zhang *et al*. reported that p53 facilitates ferroptosis in ovarian cancer cells treated with iron oxides^[76]^.

Sixth, a tight interplay between ferroptosis and translation has attracted increasing attention in the field of cancer research. Translation initiation machinery, such as initiation factors and ribosomal proteins, modulates gene regulation during nutrient deprivation and metabolic stress^[78]^. The upregulation of mammalian target of rapamycin (mTOR), a master regulator of translation initiation, results in the increased expression of cancer-promoting genes such as eIF4E, a limiting factor for translation initiation in most cancers, including ovarian cancer^[79,80]^. The activation of mTOR leads to the phosphorylation of eukaryotic translation initiation factor 4E-binding protein (4E-BP), increased recruitment of eukaryotic translation initiation factor 4E (eIF4E), and initiation of protein translation^[81]^. eIF4E plays a key role in many physiological processes such as protein synthesis, cell growth, proliferation, angiogenesis, and carcinogenesis. eIF4E was found to inhibit aldehyde dehydrogenase (ALDH) activity and increase the ferroptosis sensitivity of ovarian cancer cells by accumulating lethal lipid peroxidation^[82,83]^. The ALDH enzyme family detoxifies ROS-mediated lipid peroxidation-generated aldehydes such as MDA. In addition, the stimulation of GPX4 protein synthesis was enhanced through the mTOR/eIF4E axis^[84,85]^. Therefore, the dysregulation of translational machinery alters susceptibility to ferroptosis in ovarian cancer cells. Collectively, the mTOR signaling pathway is involved in many crucial biological processes, including ferroptosis, as it is frequently activated in a wide range of tumors, including ovarian cancer.

Finally, cancer cells require not only glucose and glutamine metabolism in the tumor microenvironment, but also iron metabolism to maintain cell survival^[9]^. Iron plays an important role in ferroptosis, and ovarian cancer is characterized by high intracellular iron content^[6]^. Ovarian cancer cells upregulate transferrin receptor (the iron importer) and downregulate ferroportin (the iron efflux pump), indicating increased iron uptake^[7]^. That is, cancer cells increase iron supply and decrease iron loss based on a unique phenotype of iron metabolism^[9]^. Therefore, a literature search was performed to determine whether the metabolic pathway characteristic of cancer cells affects iron transport and iron metabolism associated with ferroptosis. Only one paper showed that glyceraldehyde-3-phosphate dehydrogenase, a key enzyme in glycolysis, is involved in iron metabolism as a transferrin-binding protein, independent of its canonical role in glycolysis^[86]^. Iron plays an important physiological role, including oxygen transport and energy metabolism, but little is known about whether energy metabolism affects iron homeostasis in ovarian cancer.

## THERAPEUTIC STRATEGY BASED ON FERROPTOSIS

Despite the advances in the treatment of ovarian cancer, many patients experience intrinsic and acquired resistance to anticancer drugs, which have poor outcomes. Several recent in vitro and animal studies have shown that ferroptosis inducers, chemotherapy, and immunotherapy can synergistically affect ovarian cancer^[35,41]^. In this study, two potential treatment options targeting ferroptosis were investigated: The first treatment option directly induces ferroptosis and its downstream pathways, and the other targets metabolic vulnerabilities associated with ferroptosis, such as glycolysis, glutaminolysis, and energy stress dependence. The key molecules associated with the former include xCT, GSH, GPX4, intracellular labile iron, and ferroptosis-inhibitory pathways. Cancer has evolved several regulatory mechanisms of ferroptosis, which neutralize ROS and prevent lipid peroxidation, including the xCT-GSH-GPX4 axis^[15,42,44]^, ferroptosis-suppressor-protein 1 (FSP1)-coenzyme Q10 (CoQ10)-NADPH axis^[87]^, and Hippo pathway^[88]^. Several studies have also reported on xCT molecular-targeted therapy for in vivo application. Considering that highly sensitive xCT inhibitors and cystine deprivation can induce ferroptosis, several ferroptosis-inducing agents have been developed^[15,35,42]^. Some recent reviews have provided evidence for the association between ferroptosis inhibition and ovarian cancer progression, discussing the potential therapeutic application of ferroptosis-inducing agents^[15,35,42-44]^. On the contrary, the latter is a therapeutic strategy that induces ferroptosis by targeting rewired energy metabolism and its potential metabolic compensation^[31,41]^. The suppression of specific metabolism may represent an attractive therapeutic strategy for the treatment of ovarian cancer^[41]^. Therapeutic strategies targeting the ferroptosis pathway and metabolic vulnerabilities associated with ferroptosis have been reported in breast cancer and other types of cancer^[13,25]^. In this review, therapeutic strategies were divided into “a therapeutic strategy focusing on the ferroptosis pathway” and “a therapeutic strategy focusing on metabolic vulnerability and nutrient addiction.” However, mechanisms such as “disulfide stress” do not apply to either strategy.

### Therapeutic strategy focusing on the ferroptosis pathway

SLC7A11, a molecule that protects cancer cells from ferroptosis-induced cell death, is overexpressed in different types of cancers, including ovarian cancer, lung cancer, triple-negative breast cancer, PDAC, renal cell carcinoma, liver cancer, and glioma, and it is associated with aggressive phenotypes and poor prognosis^[16,46,89]^. Ferroptosis resistance involves the sustained overexpression of xCT and activation of its downstream signaling. The upregulation of xCT induces oxidative stress resistance and protects against lipid peroxidation^[90]^. xCT inhibitors have drawn increasing attention because of their antitumor effect on ovarian cancer using preclinical animal models^[91]^. Class 1 ferroptosis inducers, including erastin, imidazole ketone erastin, sulfasalazine, sorafenib, and HG106, directly inhibit xCT activity^[92,93]^ and induce ferroptosis by preventing cystine uptake and depleting cysteine or GSH, thereby inducing lipid peroxidation and cell death^[15,49]^. Several studies have highlighted the importance of elastin in single or combination therapeutic strategies for ovarian cancer. For example, C-Myc amplified in ovarian cancer cells inhibits ferroptosis by inducing NCOA4-mediated ferritinophagy^[94]^, but erastin selectively kills iron-addicted ovarian cancer cells by inducing ferroptosis and promoting NCOA4-mediated ferritinophagy and mitochondrial dysfunction^[95]^. However, ovarian cancer cells with low intracellular iron pools are resistant to erastin treatment^[95]^, indicating that iron levels can determine cell sensitivity to ferroptosis. Ovarian cancer cells that are less susceptible to ferroptosis may be platinum-resistant^[96]^ and may be clinically recommended for co-treatment with ferroptosis-inducing agents. Erastin synergizes with cisplatin to inhibit ovarian cancer growth through ferroptosis^[97]^. Erastin sensitizes ovarian cancer cells to wee1 inhibitor AZD1775 and synergistically inhibits their growth^[98]^. Wee1 is a G2 checkpoint kinase. PARP inhibitors were reported to promote SLC7A11-mediated ferroptosis^[99]^. However, erastin can facilitate ovarian cancer cell invasion in vivo by inducing IL-8 production and then M2 macrophage polarization^[100]^. Despite the antitumor effect of erastin, evidence has also shown that iron concentration and macrophage polarization in the tumor microenvironment promote the resistance of ovarian cancer cells to erastin-induced ferroptosis. Although xCT inhibition as a regulator of ferroptosis is a potential strategy for cancer therapy, the potential targets of ferroptosis in the treatment of ovarian cancer in vivo and their mechanisms remain poorly understood. Furthermore, Ras-selective lethal 3 (RSL3) and the 5,6-dihydro-2H-pyrano[3,2-g]indolizine (DPI) class of luminogen (DPI, also known as ML162) are known as class 2 ferroptosis inducers that directly inhibit GPX4 enzymatic activity^[91,93]^. RSL3 induces ferroptosis by inhibiting the GPX4 activity in drug-resistant ovarian cancer cells^[101]^. Apart from class 1 and class 2 ferroptosis inducers, targeting molecules associated with ferroptosis in ovarian cancer is an emerging field of therapeutics.

In addition, ferroptosis is characterized not only by the loss of GPX4 activity and subsequent accumulation of labile iron and excessive ROS production, but also by the peroxidation of polyunsaturated fatty acids (PUFAs)^[102,103]^. The inhibition of GPX4 causes the aberrant accumulation of PUFA hydroperoxides. In general, acyl-CoA synthetase long-chain family member 4 (ACSL4), lysophosphatidylcholine acyltransferase 3 (LPCAT3), and lipoxygenases (e.g., 15-LO) promote the peroxidation of phospholipids containing PUFA during ferroptosis^[104]^. Zhao *et al*. summarized several inhibitors targeting different enzymes in the lipid metabolism network (e.g., ACC, fatty acid synthase, sterol regulatory element-binding protein 1, and stearoyl-CoA desaturase) and discussed targeting lipid metabolism in ovarian cancer^[104]^. Therefore, the role of lipid peroxidation in ferroptosis in several human cancers, including ovarian cancer, has been emphasized^[44,104]^. Targeting lipid metabolism may be an important and potential strategy in cancer therapy; thus, combining drugs that modulate ferroptosis with conventional cancer therapies has received significant interest^[104]^. Table 1 shows the agents that affect the ferroptosis pathway by modulating the accumulation of iron, ROS, and lipid peroxides.

**Table 1:** Agents that affect the ferroptosis pathway by modulating the accumulation of iron, ROS, and lipid peroxides

**Agents**		**Function**	**Ref.**
**The biological function or action of the agents**	**Official symbol/Official full name**		
**The accumulation of iron**			
NRF2 inhibition	NRF2/Nuclear factor erythroid 2-related factor 2	NRF2 decreases the labile iron pool and increases ferroptosis resistance through controlling HERC2 [HECT and RLD domain containing E3 ubiquitin protein ligase 2; E3 ubiquitin ligase for NCOA4 and FBXL5 (F-box and leucine-rich repeat protein 5)] and VAMP8 (vesicle-associated membrane protein 8; mediating autophagosome-lysosome fusion).	[105]
HMOX1 inhibition	HMOX1/Heme oxygenase 1	HMOX1 is the enzyme responsible for degradation of heme and generates antioxidant and anti-inflammatory byproducts. Upregulation of HMOX1 inhibits ferroptosis and promotes ovarian cancer cell growth.	[106]
Ferritinophagy/Autophagic degradation of ferritin		Ferroptosis vulnerability is induced through autophagic degradation of ferritin (i.e., ferritinophagy) of ferritin heavy chain 1 (FTH1) in cisplatin-resistant ovarian cancer.	[107]
Iron nitroprusside		Iron nitroprusside is thought to be an effective treatment for ovarian cancer because it induces lipid peroxidation via the Fenton reaction and subsequently promotes ferroptosis.	[45]
**The accumulation of ROS**			
SLC7A11 degradation	HRD1/E3 ubiquitin ligase 3-hydroxy-3-methylglutaryl reductase degradation	Promoting ubiquitination-dependent SLC7A11 degradation	[108]
FZD7 inhibition	FZD7/Wnt receptor Frizzled 7	FZD7 reduces ferroptosis sensitivity through the β-catenin-Tp63-GPX4 pathway in platinum-resistant ovarian cancer cells.	[109]
FSP1 inhibition	FSP1/Ferroptosis suppressor 1	FSP1 is a GSH-independent suppressor of ferroptosis that acts as an NADH-dependent CoQ10 oxidoreductase, contributing to ferroptosis resistance via reducing CoQ10.	[15]
The Hippo effectors	YAP/Yes-associated protein 1; TAZ/Transcriptional coactivator with PDZ-binding motif	The Hippo effectors YAP and TAZ induce ferroptosis in ovarian cancer cells through overexpressing Angiopoietin-Like 4 (ANGPTL4) and NADPH Oxidase 2 (NOX2). The Hippo proteins control cell fate.	[110]
NRF2 modulator	NCTD/Norcantharidin	NCTD, a normethyl compound of cantharidin, facilitates ferroptosis by inhibiting the NRF2/HO-1/GPX4/xCT axis.	[111]
**The accumulation of lipid peroxides**			
SCD1 inhibition	SCD1/Stearoyl-CoA desaturase 1	SCD1 catalyzes the formation of monounsaturated fatty acids (MUFAs), specifically oleate and palmitoleate. Inhibition of SCD1 causes iron-mediated lipid peroxidation and mitochondrial dysfunction by downregulating GPX4 and then induces ovarian cancer cell death. SCD1 inhibitor co-treatment may enhance the antitumor efficacy of ferroptosis inducers in ovarian cancer.	[112-114]
ACSL1 inhibition	ASCL1/Acyl-CoA synthetase long-chain family member 1	ACSL1 reduces the level of lipid peroxidation and enhances ferroptosis resistance in ovarian cancer through increasing the stability of FSP1.	[115]
**Others**			
Ferroptosis-related gene	PRNP/Prion protein	Overexpression of ferroptosis-related gene prion protein (PRNP) inhibits ovarian cancer proliferation and invasion.	[116]
Ropivacaine		A local anesthetic ropivacaine induces ferroptosis in ovarian cancer cells by inactivating the PI3K/Akt pathway.	[117]

GPX4: Glutathione peroxidase 4; SLC7A11: solute carrier family 7 member 11.

### Therapeutic strategy focusing on metabolic vulnerabilities and nutrient addiction

In response to dynamically changing nutrient availability in the tumor microenvironment, cancer cells facilitate cellular adaptations to reprogram metabolic pathways. Therefore, metabolic vulnerabilities are attacked using the synthetic lethality approach in ovarian cancer cells overexpressing xCT, rather than directly inhibiting xCT or GPX4. Cancer cell-specific metabolic pathways or nutrient addiction, such as glycolysis, glutaminolysis, or other energy stress dependence, can also be utilized as therapeutic targets. However, it is difficult to assess in real time which metabolic pathways cancer cells are currently most dependent on. Despite recent progress in the development of xCT inhibitors, effective treatment interventions focusing on metabolic vulnerabilities remain unmet.

**Figure 2:**
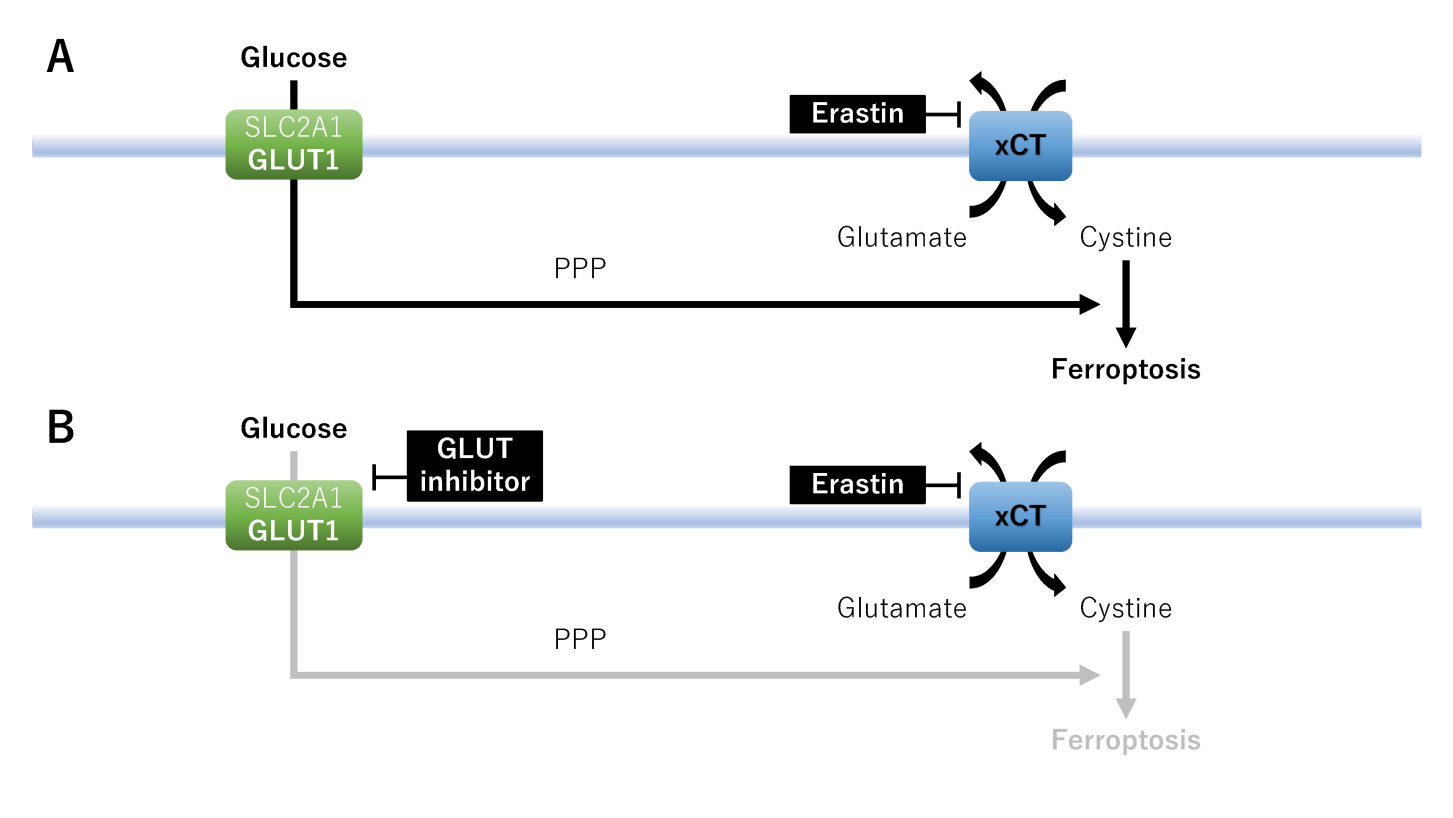
A therapeutic strategy focused on metabolic vulnerabilities and nutrient addiction: glucose metabolism. Effects of erastin on ferroptosis in the absence (A) or presence (B) of GLUT inhibitors. GLUT1/SLC2A1: Glucose transporter 1; PPP: pentose phosphate pathway; xCT: cystine/glutamate antiporter SLC7A11.

First, xCT inhibitors such as erastin have emerged as an effective treatment option to facilitate ferroptosis in a high-glucose tumor environment^[19]^. Ferroptosis has been reported to be dependent on glucose uptake by GLUT 1 (also known as SLC2A1)^[18]^. The maintenance of glycolysis in xCT^high^ cancer cells is essential for constituting a potential treatment strategy to induce ferroptosis [Figure 2A]. By contrast, glucose deprivation induced by GLUT inhibitors selectively blocks xCT inhibitor-induced ferroptosis, thereby suppressing cancer cell death^[18]^. That is, xCT inhibitors may exhibit anticancer properties against cancer cells with elevated GLUT1 expression in a high-glucose tumor environment, whereas glucose deprivation blocks ferroptosis-induced cell death^[18,19]^ [Figure 2B]. xCT inhibitors show contrasting effects in the presence or absence of glucose in the tumor microenvironment.

**Figure 3:**
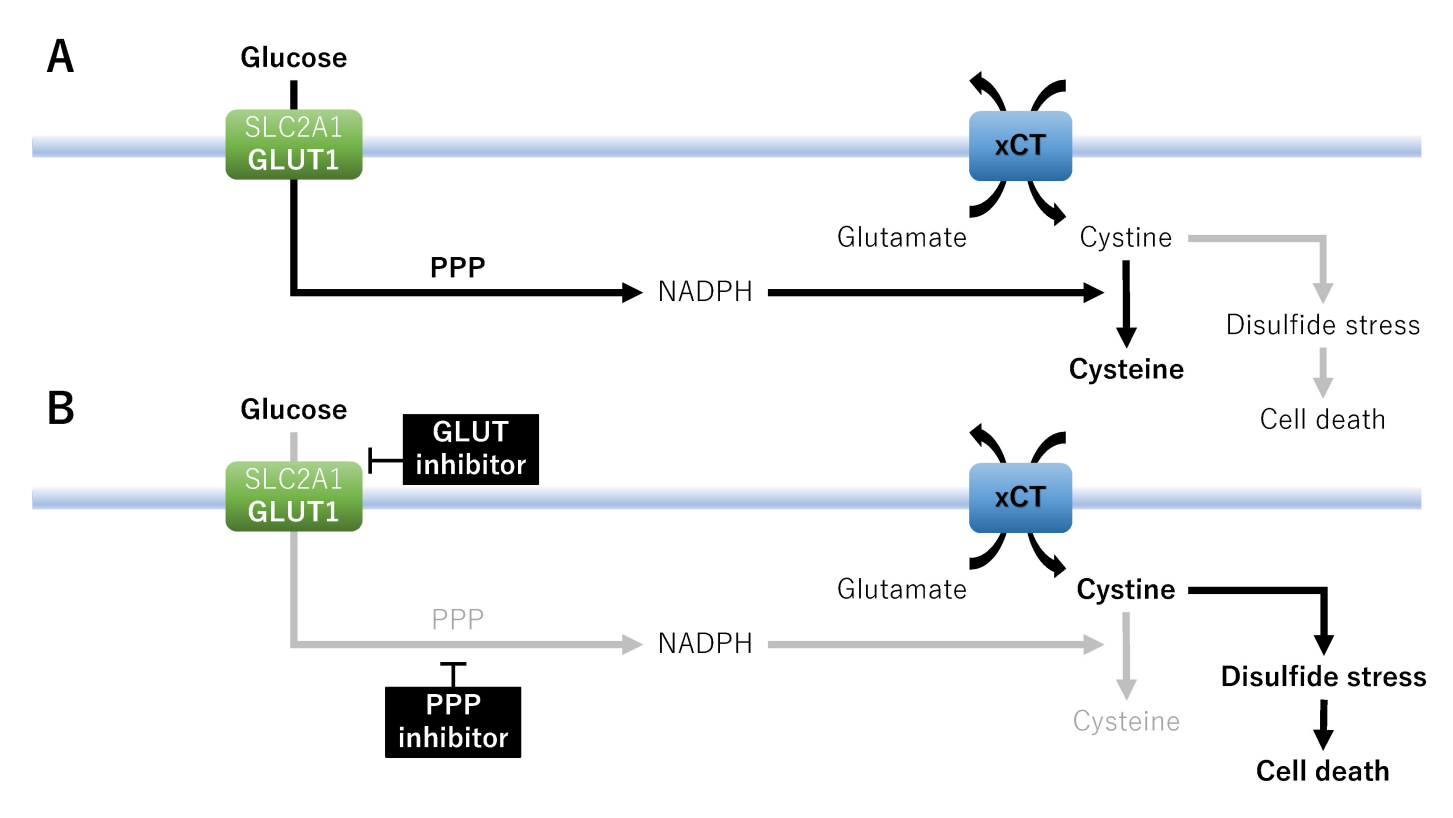
A therapeutic strategy focused on metabolic vulnerabilities and nutrient addiction: the PPP metabolism. Effects of xCT on cell death in the absence (A) or presence (B) of GLUT inhibitors or PPP inhibitors. GLUT1/SLC2A1: Glucose transporter 1; NADPH: nicotinamide adenine dinucleotide phosphate; PPP: pentose phosphate pathway; xCT: cystine/glutamate antiporter SLC7A11.

Second, the PPP-generated NADPH plays a central role in the cellular metabolic network and redox homeostasis^[14]^. NADPH can support the biosynthesis of GSH, thioredoxin, and CoQ10 to boost the antioxidant defense in cancer cells and protect cancer cells from ferroptosis and cell death^[14]^ [Figure 3A]. Therefore, targeting the PPP in tumor cells may provide a therapeutic strategy based on ferroptosis. Glucose deprivation reduces the PPP-mediated NADPH generation, making it impossible to reduce insoluble cystine imported via xCT to a more soluble cysteine, thereby inducing disulfide stress (i.e., cystine-dependent toxicity) and leading to rapid cell death^[48]^ [Figure 3B]. This finding suggests that xCT-overexpressing cancer cells are sensitive to glucose starvation-induced cell death. Furthermore, cysteine depletion in CCC abolishes glycolysis and OXPHOS and inhibits cancer cell proliferation^[118]^, indicating that cysteine depletion caused by the decreased conversion of cystine to cysteine plays a critical role in cancer therapy. Therefore, not only the GLUT inhibitor but also the glucose-6-phosphate dehydrogenase inhibitor or 6-amino-nicotinamide, a nicotinamide analog, may be used as an inhibitor of the PPP. Collectively, these studies suggest that xCT is beneficial for cancer cells by suppressing ferroptosis, while glucose deprivation and NADPH depletion caused by a reduction in carbon from glucose entering the PPP can promote cancer cell death^[26,48]^.

**Figure 4:**
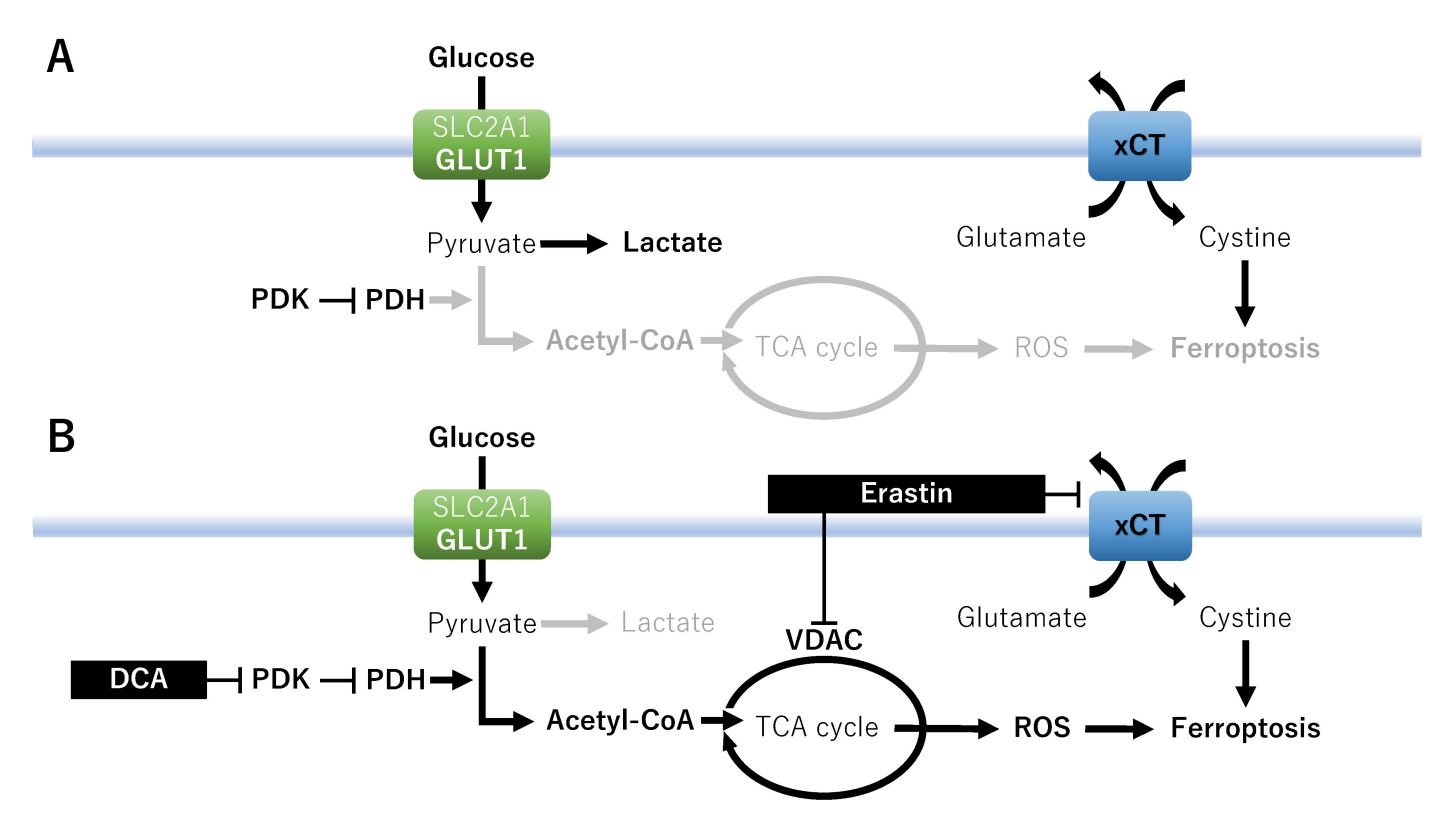
A therapeutic strategy focused on metabolic vulnerabilities and nutrient addiction: PDK-dependent metabolism. Effects of xCT on ferroptosis in the absence (A) or presence (B) of erastin or PDK inhibitor. DCA: Dichloroacetate; GLUT1/SLC2A1: glucose transporter 1; PDK: pyruvate dehydrogenase kinase; PDH: pyruvate dehydrogenase; ROS: reactive oxygen species; TCA: tricarboxylic acid; VDAC: voltage-gated anion channels; xCT: cystine/glutamate antiporter SLC7A11.

Third, PDK4 inhibits the TCA cycle and reduces the production of PUFAs to enhance ferroptosis resistance^[18]^ [Figure 4A]. Dichloroacetate, a PDK inhibitor, induces the metabolic switch from glycolysis to OXPHOS to generate ROS accumulation, thereby facilitating ferroptosis^[119]^ [Figure 4B]. Erastin can mediate ferroptosis through mitochondrial voltage-gated anion channels (VDAC) and xCT, which impairs VDAC function, thereby resulting in mitochondrial dysfunction, ROS production, and cell death^[120]^. Thus, xCT inhibitors enhance mitochondrial respiration and increase ROS production to promote ferroptosis, which may be a potential therapeutic strategy in cancer cells exhibiting metabolic reprogramming from glycolysis to OXPHOS. Furthermore, co-treatment with PDK inhibitors and erastin may synergistically enhance ferroptosis in cancer cells overexpressing GLUTs and xCT^[18]^.

**Figure 5:**
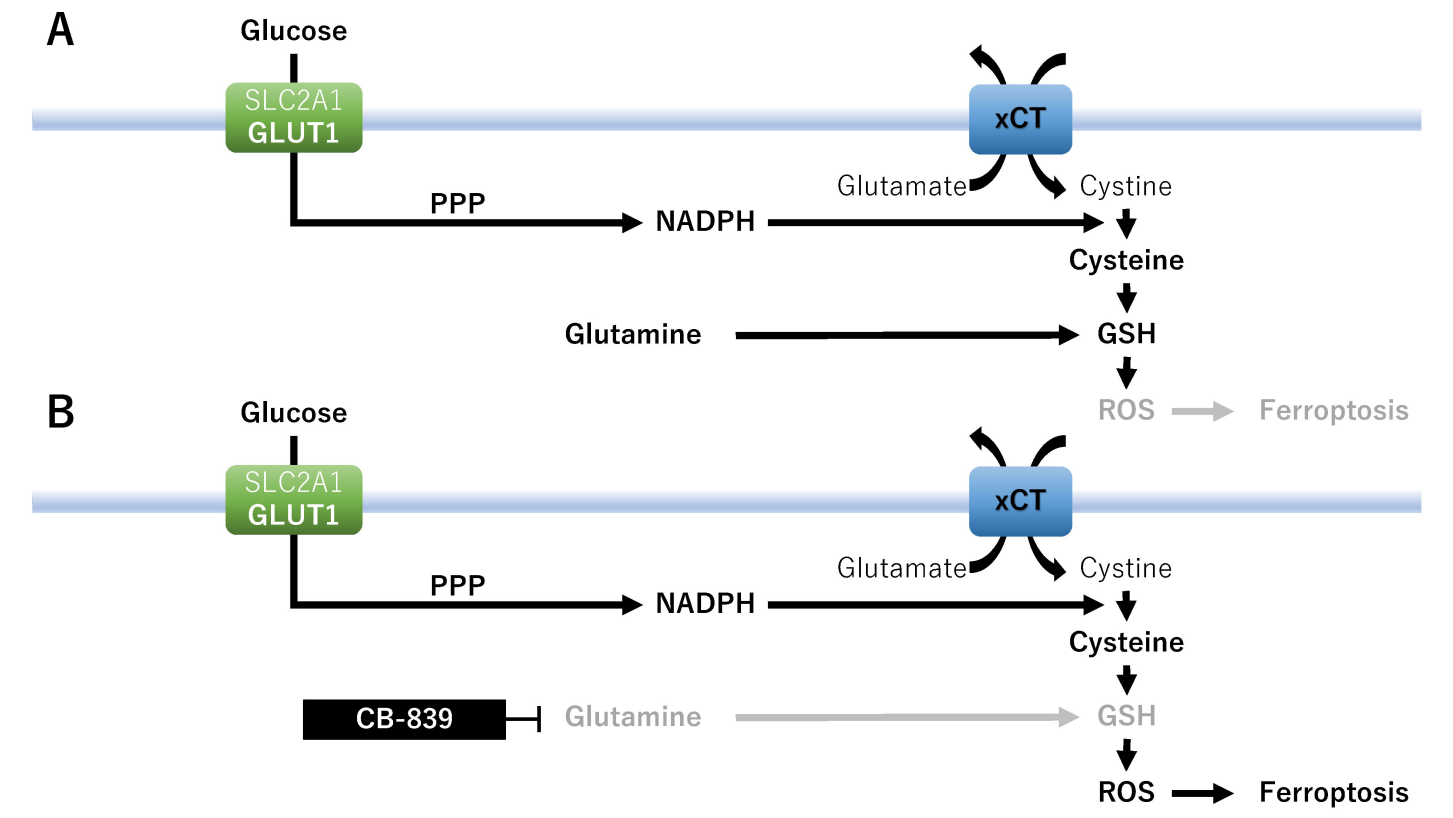
A therapeutic strategy focused on metabolic vulnerabilities and nutrient addiction: glutamine metabolism. Effects of xCT on ferroptosis in the absence (A) or presence (B) of glutaminase inhibitor. GLUT1/SLC2A1: Glucose transporter 1; GSH: glutathione; NADPH: nicotinamide adenine dinucleotide phosphate; PPP: pentose phosphate pathway; ROS: reactive oxygen species; xCT: cystine/glutamate antiporter SLC7A11.

Fourth, glutaminase mediates the conversion of glutamine to glutamate, leading to the entry of glutamine into the TCA cycle. Cancer cells upregulate and consume glutamine to produce metabolic fuel for cancer cell proliferation and redox status regulation [Figure 5A]. Cancer cells may be more sensitive to glutaminase inhibition under high glutamine import compared with that under low glutamine import^[25]^. xCT-overexpressing cancer cells are sensitive to glutaminase inhibition because the inhibition of glutamine metabolism decreases GSH production and increases ROS production^[13,121]^ [Figure 5B]. xCT not only catalyzes glutamate release, but also regulates glutamine uptake^[16]^. xCT-overexpressing cancer cells lead to glutamine dependency, which presents potential metabolic vulnerabilities as therapeutic targets. A recent review has discussed new breast cancer treatment strategies based on glutaminase modification, leading to cellular ferroptosis^[25]^. CB-839 (telaglenastat), a glutaminase inhibitor, blocks tumor glutamine consumption^[122]^. A phase 1 clinical study will assess the safety, tolerability, and preliminary evidence of the antitumor activity of CB-839 in combination with the mTOR inhibitor sapanisertib (MLN0128)^[122]^.

Finally, some drugs or natural compounds have been reported to have antitumor properties by targeting metabolic vulnerabilities such as alterations in glycolysis, OXPHOS, and glutamine metabolism^[13]^. For example, Yung *et al*. reported that dual-targeted therapy utilizing AMPK-activating drugs and vascular endothelial growth factor/programmed cell death 1 blockade may be a new treatment option for ovarian cancer focusing on metabolic vulnerability^[66]^. A natural‐occurring enzyme, glucose oxidase, converts glucose into non-metabolizable gluconic acid and H_2_O_2_, amplifying the ferroptotic damage. A bioactive protein, MAP30, isolated from bitter melon seeds, triggered apoptosis and ferroptosis in various ovarian cancer cells^[123]^. Furthermore, research on compounds derived from natural products may lead to the development of new treatment options that promote ferroptosis.

## DISCUSSION

In this review, the regulatory mechanism of ferroptosis through multiple metabolic pathways was summarized, and therapeutic strategies targeting ferroptosis in ovarian cancer were discussed. In particular, ovarian cancer is strongly influenced by an iron- and ROS-dependent mode of cell death, namely, ferroptosis, from the early stages of carcinogenesis. Changes in iron metabolism, lipid peroxidation responses, and a wide range of energy metabolism play important roles in regulating ferroptosis sensitivity of ovarian cancer cells^[124]^. Two therapeutic strategies targeting xCT were highlighted in this review, that is, directly inhibiting xCT activity and targeting glucose/glutamine dependency as a therapeutic vulnerability in xCT-overexpressing cancer cells. New perspectives for improving cancer therapy based on ferroptosis were also discussed. xCT inhibitors (e.g., erastin) can induce cell death when carbon from glucose, glutamine, or fatty acids is abundant^[16]^. Conversely, nutrient starvation, such as glucose and glutamine, causes cancer cell death because of dysfunction of the xCT-associated antioxidant system. For example, glucose deprivation induces rapid NADPH depletion, promoting ferroptosis-mediated oxidative stress and cell death^[16]^. Glucose-depleted cancer cells may display glutamine addiction, resulting in the survival of such cells. Elevated glutamine metabolism makes cancer cells more susceptible to glutamine deficiency caused by glutaminase inhibitors, which rapidly induces cell death^[122]^. Drugs that block intrinsic and acquired nutrient addiction may promote the susceptibility of cancer cells to ferroptosis^[16,57]^. Therefore, therapeutic approaches that use nutrient addiction to target metabolic vulnerabilities may be effective in overcoming ferroptosis resistance.

Moreover, preclinical studies revealed that ferroptosis inducers contribute to the enhanced efficacy of immunotherapy^[125]^ and chemotherapy^[126]^. For example, PARP inhibitors sensitize ovarian cancer cells to ferroptosis by synergistically activating ATM/ATR and causing DNA damage^[126]^. The enhancement of cancer efficacy by eliminating drug resistance may be achieved through synergistic combinations of ferroptosis and existing therapeutic approaches^[15]^. Thus, cancer therapy that induces ferroptosis could be an innovative therapeutic strategy in ovarian cancer^[35,41]^. However, xCT has been demonstrated to play contradictory roles in ferroptosis regulation in a tumor-promoting or suppressive manner, depending on changes in energy and nutrient metabolic pathways^[48,57]^. xCT functions as a double-edged sword to modulate various types of cancer survival and death by regulating the redox balance, nutrient dependency, energy stress, and ferroptosis process^[16]^. Furthermore, the role of metabolic flexibility and vulnerability in regulating xCT-mediated ferroptosis was summarized, and the current understanding of ferroptosis-induced therapy in ovarian cancer was discussed. Ferroptosis inducers should be used on the basis of the metabolic characteristics of cancer cells.

## CONCLUSION AND FUTURE PERSPECTIVES

Cells have evolved mechanisms to maintain redox homeostasis through metabolic reprogramming whenever they encounter a large burden of oxidative stress resulting from changes in the microenvironment^[127]^. Cancer cells are forced to alter their energy and nutrient metabolic pathways to adapt to ever-changing environmental changes^[127]^. Cancer cells acquire autonomous capabilities for tumor promotion by upregulating xCT activity, inducing antioxidant defenses, and suppressing ferroptosis^[16,46]^. The key molecules in signaling pathways associated with glycolysis (e.g., PDK4), OXPHOS (e.g., IDH1 and αKG), glutaminolysis (e.g., glutaminase), PPP (e.g., G6PD and NADPH), and P53 pathway are critically involved in regulating ferroptosis in ovarian cancer^[12]^. xCT-overexpressing cancer cells depend on glucose, glutamine, and fatty acids for an energy source to acquire proliferative and survival advantages^[16,17,19,68]^. All main metabolic pathways, including glycolysis, TCA cycle, glutaminolysis, OXPHOS, and PPP, are individually altered in ovarian cancer cells^[13]^.

Several reliable molecular biomarkers can predict cell death associated with ferroptosis. However, the biopsy of tumor tissue has its own limitations. Liquid biopsy provides a minimally invasive diagnostic modality to assess the molecular characterization of the tumor and to allow a personalized approach to patients with effective treatment in real time^[128]^. Liquid biopsy of the blood and peritoneal fluid in patients with recurrent ovarian cancer may be used as a drug screening platform to select potential drugs. The gene expression profile can be verified by reverse transcription-polymerase chain reaction assay using customized pre-selected genes. This panel should include candidate genes potentially associated with ferroptosis, for example, SLC7A11, GLUT1, PDK, PDH, glutaminase, GPX, BECN1, ACC, and AMPK. xCT inhibitors can also have therapeutic benefits for ovarian cancer cells growing in a high-glucose tumor environment (e.g., ovarian cancer cells overexpressing SLC7A11, GLUT1, PDK, glutaminase, and GPX). By contrast, drugs that target metabolic pathways (e.g., GLUT1 inhibitors, PPP inhibitors, PDK inhibitors, and glutaminase inhibitors) may provide promising efficacy in ovarian cancer cells harboring nutrient addiction. Therefore, drugs can be selected on the basis of the expression pattern of ferroptosis-inducing genes (e.g., SLC7A11, GSH, and GPX) or genes associated with metabolic pathways that affect ferroptosis (e.g., GLUT, PDK4, glutaminase, VDAC, and G6PD). Such gene expression patterns may serve as biomarkers for selecting patients with cancer for personalized treatment. Further studies based on the regulation of xCT expression, ROS stress and redox homeostasis, and energy stress caused by specific nutrient addiction or deficiency will increase the clinical importance of ferroptosis modulation as an effective therapeutic strategy for ovarian cancer.

## DECLARATIONS

### Copyright

© The Author(s) 2023.
